# Telemedicine for Kidney Transplant Recipients: Current State, Advantages, and Barriers

**DOI:** 10.1097/TP.0000000000004660

**Published:** 2023-06-02

**Authors:** Bartu Hezer, Emma K. Massey, Marlies E.J. Reinders, Mirjam Tielen, Jacqueline van de Wetering, Dennis A. Hesselink, Martijn W.F. van den Hoogen

**Affiliations:** 1 Erasmus MC Transplant Institute, University Medical Center Rotterdam, Department of Internal Medicine, Rotterdam, the Netherlands.

## Abstract

Telemedicine is defined as the use of electronic information and communication technologies to provide and support healthcare at a distance. In kidney transplantation, telemedicine is limited but is expected to grow markedly in the coming y. Current experience shows that it is possible to provide transplant care at a distance, with benefits for patients like reduced travel time and costs, better adherence to medication and appointment visits, more self-sufficiency, and more reliable blood pressure values. However, multiple barriers in different areas need to be overcome for successful implementation, such as recipients’ preferences, willingness, skills, and digital literacy. Moreover, in many countries, limited digital infrastructure, legislation, local policy, costs, and reimbursement issues could be barriers to the implementation of telemedicine. Finally, telemedicine changes the way transplant professionals provide care, and this transition needs time, training, willingness, and acceptance. This review discusses the current state and benefits of telemedicine in kidney transplantation, with the aforementioned barriers, and provides an overview of future directions on telemedicine in kidney transplantation.

## Graphical abstract

**Figure:**
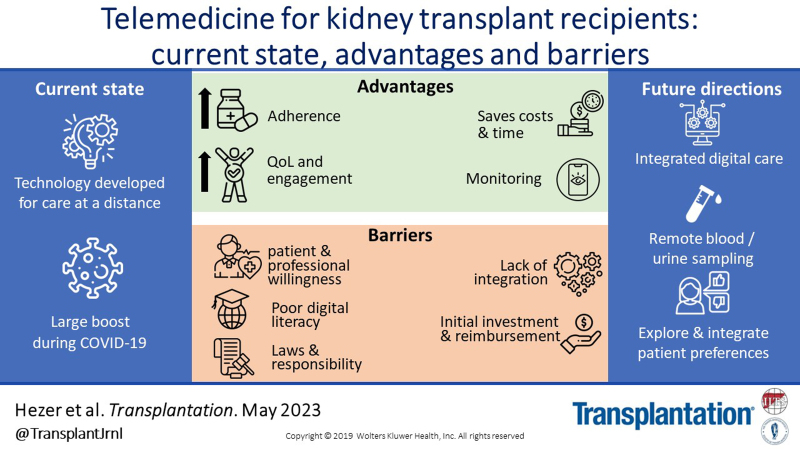

## INTRODUCTION

**FIGURE 1.**
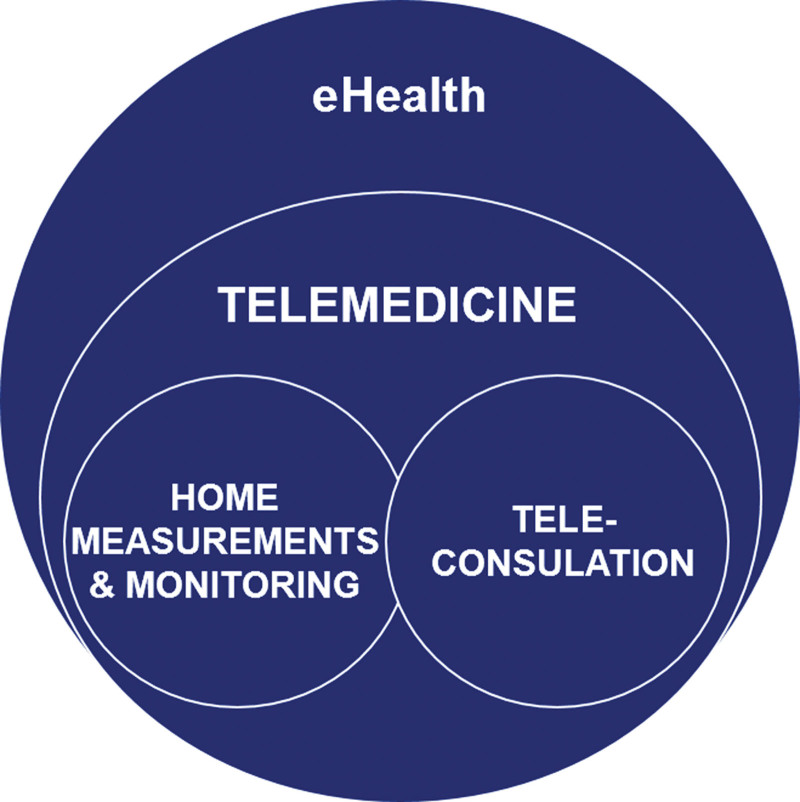
An illustration of relationships eHealth, telemedicine, teleconsultation, and home measurements and monitoring.

Traditionally the interaction between transplant recipient and healthcare provider has been in-person. With increasing use of information and communication technologies in the community, these technologies have impacted the delivery of healthcare and led to concept of eHealth, defined by the World Health Organization as the use of information and communication technologies in support of health and health-related fields.^1^ Telemedicine can be defined as the use of electronic information and communication technologies to provide and support healthcare when distance separates the participants.^2^ This can encompass, for example, home measurements and monitoring of symptoms as well as the use of teleconsultations (as illustrated in Figure 1).

Essential in the concept of telemedicine is the delivery of care at a distance with a patient following their daily routines and healthcare provider situated elsewhere. One of the first areas in medicine to implement telemedicine was diabetes care, in which measuring glucose levels at home, work, or elsewhere is crucial for the patient to receive the correct therapy (insulin). Although implemented first in metropolitan areas, this was later adopted in other geographical areas.^3^ Patients communicate frequently with their healthcare provider, often by phone or email, to discuss and adjust the therapy. The field of diabetes has undergone enormous progress during recent years, with new technologies (continuous and flash glucose measurements), new analysis (time in range, estimated hemoglobin A1c), and rapid transfer of data (web-based platforms, like LibreView).^4^ In other patient groups, including patients with chronic kidney disease, relatively simple technology, such as cell phones with text messaging interventions, has shown significant improvements in compliance with medicine taking, asthma symptoms, stress levels, smoking cessation rates, self-efficacy, a reduction in (intradialytic) weight gain, and sodium intake.^5-7^

Telemedicine for solid organ transplantation is a relatively new area. In this article, we will review the current state of telemedicine for kidney transplants recipient. We discuss the state of the art and advantages of integrating telemedicine into everyday care for kidney transplant recipients and address barriers and limitations for successful implementation. Finally, we provide an overview of future directions for telemedicine in transplantation.

## STATE OF ART AND BENEFITS OF TELEMEDICINE FOR THE KIDNEY TRANSPLANT RECIPIENT

Although the earliest publications on telemedicine in nephrology date back to the early 1990s with special video connections for dialysis facilities in central Texas,^8^ reports specifically addressing telemedicine and kidney transplantation date from the early 2010s. Groundbreaking work has been done by McGillicuddy et al from the Medical University of South Carolina. In a plethora of publications, they have reported on different aspects of telemedicine incorporated into the care for kidney transplant recipients.^9-16^ In Table 1, their work and the work of other groups are summarized chronologically and discussed per topic below.

**TABLE 1.** Studies on various topics of telemedicine for kidney transplant recipients, in chronological order

Author	Year	No. of patients	Topics	Design/objective	Instruments	Results	Concerns
Connor et al^17^	2011	123	Chronic care	Case study, description of current practice BP and weight taken at home or at local hospital Blood test taken beforehand	Telephone consultation 3-monthly over a 3-y period with only once a year face-to-face contact	Two patients preferred face-to-face only. No report of patient safety issues In 30 patients, a mean of 39 km and 8 kg CO_2_-equivalent saved per teleconsultation	No data on adherence to telephone consultation nor full economic analysis Loss of visual clues to a patient’s well-being Service is only offered to patient with 12-mo stable graft function
McGillicuddy et al^9^	2013	19	Medication adherence BP monitoring	Proof-of-concept randomized controlled trial (3 mo follow-up) to assess patient and provider acceptability, feasibility of mobile phone–based remote monitoring	Smartphone (Motorola Droid X) Wireless smartphone-enabled) medication tray (Maya MedMinder) Wireless (Bluetooth-enabled) BP monitor (Fora D15b).	Instruments safe, highly acceptable, and useful to patients and providers At 3 mo, significantly better medication adherence (94% vs 57%) and lower systolic BP (122 vs 139 mm Hg)	Limited inclusivity (of 55 approached patients, only 20 participated and were randomized) Costs ($45 a mo) 23% device failure (Maya MedMinder)
Aberger et al^18^	2014	66	BP monitoring	Management of BP in kidney transplant recipients, single-arm study	BP monitor (model UA-767PC; A&D Medical, San Jose, CA) Upload to home computer and Good Health Gateway Patient Portal	Significant reductions in average systolic and diastolic BP (6.0 and 3.0 mm Hg, respectively) after 30 d	Lack of computer access Limited computer literacy Patient forgetfulness, apathy, or motivational decline over time Obesity (cuff size) Lack of understanding of importance of blood pressure management
McGillicuddy et al^11^	2015	18	BP monitoring	Follow-up (12 mo posttrial) of the above-mentioned proof-of-concept trial^9^	See above^9^	Lower systolic BP in eHealth group (132 vs 154 mm Hg) sustained for 12 mo posttrial	None, but more former eHealth users reported using various methods to assist adherence
Schmid et al^19^	2017	46	Medication adherence Chronic and acute care	Randomized trial comparing standard posttransplant care with telemedicine-supported case management	Touch screen monitors (non-mobile), real-time video consultation, combined with telemedical education, support, and coaching	At 1 y less nonadherence in the telemedicine group (17% vs 57%) and less acute unplanned hospitalization (median 0 vs 2) and shorter hospital stays (median 0 vs 13 d)	Extra staff needed (50% part-time transplant nurse), but cost-effective No standard reimbursement of costs Limited to living donor kidney recipients Data protection laws prohibited tablets with software for mobile telemonitoring
Levine et al^20^	2019	108	Medication adherence	Cohort study	Mobile app Transplant Hero and Pebble Smart watch Technology	No difference in coefficient of variability of tacrolimus (32%–36%)	No adherence to technology was analyzed
Han et al^21^	2019	138	Medication adherence	Randomized trial comparing adherence (BAASIS and the VAS) via mobile app vs standard of care (education)	Mobile app (Adhere4U for android) for medication management with visual and auditory reminders The app also included education on immunosuppression	At 6 mo no difference in rate of nonadherence (app group, 65% vs 62%; OR 1.14; 95% CI, 0.53-2.40) No difference between BAASIS and VAS	Low rate of patient engagement; app use only 12% at 6 mo Patients <1 y posttransplant were excluded, as were patients aged >70 y In all of the 1163 eligible patients, 138 were randomized
Udayaraj et al^22^	2019	168	Chronic and acute care	Plan do study act for a teleconsultation over telephone services with home BP measurements	Not described	Less nonattendance compared with face-fo-face (6.9% vs 2.9%) 98% surveyed patients (n = 97) were satisfied with teleconsulting A mean of 36.4 miles saved on motorized travel Saving of £6060 in total (excluding external blood sampling)	Ordering, cost, and availability of blood tests during teleconsultation No definitive cost-benefit analysis, including all healthcare-related cost
Andrew et al^23^	2020	45	Chronic care	Benefits of telehealth on patient-centered outcomes	Care provided via a telehealth platform (Health Direct Videocall) on any device and a dedicated support team	95% of patients felt that telehealth was comparable with face-to-face consultation Telehealth saved patients a total of 203 202 km in travel distance, 2771 h car travel time, and approximately Australian $31 048 in petrol costs	Unknown percentage of suitable patients No transfer of data on BP/heart rate and weight Some observations are taken by general practitioners or nurses at local hospitals
Varsi et al^24^	2021	18	Chronic care	Benefits and challenges from the perspectives of patients and healthcare providers of video consultation	Video consultation (Norwegian Health Network Cisco meeting application) on PC, tablet, or smartphone Measure own weight and BP, instrument not specified	Main benefits: reduced travel time and costs, less focus on being chronically ill	Reoccurring technical challenges Necessity to go to hospital to have blood samples drawn
Lambooy et al^25^	2021	64	Chronic care	Single-center, prospective, 2-y longitudinal, case–control study on feasibility, sustainability, and clinical outcomes of telehealth videoconferencing	Video consultation with specific telehealth software at home or at a nearest healthcare facility Both transplant (n = 32) and nontransplant chronic kidney disease patients (n = 32)	Uptake at y 1 was 71%, declined significantly to 50% in y 2 No significant differences in creatinine, BP, mortality, or hospitalization were observed between groups Great reduction in travel distance (–48% in y 1, –37% in y 2)	Decrease in uptake between y 1 and 2 Reasons uncertain, but probably divers This was not explored in the study Reimbursement and regulation remain central to the uptake and acceptance
Gonzales et al^15^	2021	136	Medication adherence and safety	Randomized, controlled trial for 12 mo, with use of mobile health-based application vs traditional care	Smartphone-enabled mobile health app (custom-made) with automatically updated medication list, reminders, and automated messages for missed doses, side-effect tracking, and home-based BP and glucose monitoring	Lower risk of medication errors (RR 0.39; 95% CI, 0.28-0.55), grade 3 adverse events (RR 0.55; 95% CI, 0.30-0.99), and rate of hospitalization (RR 0.46; 95% CI, 0.27-0.77)	Intensive pharmacist-led medication therapy monitoring instead of self-monitoring Limited inclusivity (of the 774 eligible patients, only 136 were randomly assigned)
Melilli et al^26^	2021	90	Medication adherence	Prospective, observational, multicenter, 2-phase pilot study in kidney and liver transplant recipients	TYM, a novel mHealth technology with a Quick Response code-scan app	68% used TYM regularly. 6-mo total correct intakes ranged between 69% and 76%, 12%–19% intakes were out-of-time, and 9%–12% were missed At 1 y, 53 (59%) patients were still active users of TYM	Limited eligibility of 90 of 204 patients mostly because of not owning smartphone/using apps Laborious for patients and healthcare provider No control group

BAASIS, Basel Assessment of Adherence to Immunosuppressive Medication Scale; BP, blood pressure; CI, confidence interval; OR, odds ratio; PC, personal computer; RR, risk ratio; TYM, TrackYourMed; VAS, visual analog scale.

One of the topics in which the effect of telemedicine has been studied is adherence to medication or healthcare appointments. Adherence to medication is of vital importance to maintaining good graft function, and nonadherence is highly prevalent (approximately 30% at 1 y posttransplantation).^27,28^ In 2010, it was suggested that adherence may be enhanced by the use of technology (internet-based and cellphone interventions, with voice and text messaging) to remind transplant recipients about their medications.^29^ Many different approaches of telemedicine in kidney transplantation (smartphone with medication tray, non–mobile touch screen monitors, real-time video consultation, mobile health apps, and pharmacist-led addition to technology) were operationalized in the years that followed, and irrespective of the approach, most, but not all, publications report increased adherence to medication, fewer medication errors, less variability in tacrolimus trough levels, rejection, and better attendance at (digital) healthcare appointments.^9,15,19-21,26^ This conclusion is in accordance with a 2015 systematic review on effectiveness of eHealth apps in patients with other chronic diseases (like diabetes, cardiovascular disease, and chronic lung diseases).^30^ However, in all these studies, the use of technology was combined with advanced care for adherence, and it remains unclear how much of these effects result from the technology per se. A comparable conclusion was drawn in a recent systematic review and meta-analysis on eHealth interventions to promote adherence for transplant recipients.^31^ A recent article by Hooper et al^32^ not only confirms that a bundle of interventions can effectively promote adherence but also results in a significant reduction of rejection incidence. However, a simple technology like sending mobile text message reminders can significantly increase the rate of attendance at healthcare appointments compared with sending no reminders (risk ratio 1.14; 95% confidence interval, 1.03-1.26).^33^

Other publications focus more on other topics of telemedicine in kidney transplantation, like home measurements, and the provision of digital care. After transplantation, frequent monitoring is imperative to detect allograft rejection, adjust (immunosuppressive) medication, and manage complications. Not only are hospital visits frequent after transplantation, but expertise in transplant care is also often concentrated in academic hospitals. This centralization results in long commuting times and costs for patients. Telemedicine aims to increase efficiency for both patient and provider who shifts resources to monitoring at a distance. One of the first articles on telephone and teleconsultation to provide chronic care for transplant recipients, instead of face-to-face contact,^17^ demonstrated safety of the concept, and additional studies demonstrated comparable results: reduced travel time, travel costs, and associated environmental benefits.^11,17,18,22-25^

The telemedicine program by Schmid et al^19^ at the Medical Center-University of Freiburg also deserves a special mention. In this program, telemedicine was interwoven with intensive case management to improve patient care after living donor transplantation. This led to a reduction in length and number of unplanned admissions, reducing costs significantly. It also led to an impressive improvement in disease-specific quality of life and return to employment. The authors note that their swift support and targeted actions helped to avoid more serious complications. In a later publication, the same group did an extensive analysis of the financial impact of their telemedicine program.^34^ They found that standard aftercare plus additional telemedically supported case management resulted in substantially lower costs related to unscheduled hospitalizations, and if all costs were taken into account, there was a cost reduction of almost €5000 per transplant recipient. In their calculations, they took into account the cost of a dedicated nurse, internal server provision, patient-variable costs of touch screen personal computer and software licenses, and extra infrastructure license, all accounting for an average of €3000 for telemedically supported case management of a single patient. Therefore, the resulting benefit was €2000 per patient, with the program becoming profitable starting at 15 patients annually. Cost reductions, mainly because of fewer hospitalizations, were also confirmed by McGillicuddy et al^16^ with the pharmacist-led smartphone-enabled app.

Hypertension is common in kidney transplant recipients and is associated with negative effects on cardiovascular and graft health. Blood pressure control is therefore essential to reduce these negative outcomes.^35-37^ However, office blood pressure measurement has important limitations in diagnosing hypertension because of its intra- and interindividual variability. Alternatives to office blood pressure measurement are 24-h ambulatory blood pressure monitoring (ABPM) or home monitoring. A recent meta-analysis showed that ABPM discloses a high proportion of masked hypertension, uncontrolled hypertension, and white-coat hypertension.^38^ Many (American) health organizations promote the use of home monitoring of blood pressure.^39^ However, little research has been done specifically addressing the effects of home blood pressure measurement. In the aforementioned systematic review, only 4 of 42 studies compared ABPM with both the traditional office blood pressure and home blood pressure measurements.^40-43^ However, none of those studies included in the review were performed on organ transplant recipients, and none combined blood pressure control with the use of eHealth because they were performed in the pre–smartphone era. Two studies not included in the aforementioned review used telemedicine in blood pressure measurement, specifically in kidney transplant recipients.^9,11,18^ The group of McGillicuddy used a smartphone and wireless (Bluetooth-enabled) blood pressure monitor and reported significantly lower blood pressure compared with usual care with office blood pressure measurements (122 versus 139 mm Hg), which was sustained 1 y after their proof-of-concept trial (132 versus 154 mm Hg). The study by Aberger et al^18^ confirmed lower blood pressure in a telemedicine blood pressure measurement group (values uploaded via a home computer); however, this was a single-arm group lacking a control group. These studies suggest that home monitoring may contribute to more correct interpretation of blood pressure among transplant patients.

Although home monitoring of physical measurements might eliminate the need to visit the clinic, this gain is eroded if blood and urine samples (in kidney transplant recipients, especially kidney function, proteinuria, and immunosuppressive drug levels) cannot be taken at home or locally. Home-based point-of-care creatinine measurements are available (StatSensor Xpress-i), but in a recent evaluation, they lack diagnostic accuracy for single measurements (compared with venipuncture or hospital-based point-of-care creatinine measurements) but could be useful in monitoring trends of kidney function.^44,45^ A technique that has become available in recent years is the dried blood spot (DBS) method, whereby a drop of capillary blood is collected on a filter paper. After the DBS has been sent to the healthcare center and is analyzed, dose adjustments can be made.^46,47^ However, currently, methods for urine testing at home are available (dipsticks or advanced point-of-care measurement) without direct, easy transfer of the results to the transplant center.^48^ Therefore, it is not surprising that none of the studies described in Table 1 incorporates home measurements of blood or urine.

With decreased in-hospital care and an increase in home-centered care, patients may experience a shift in responsibility and engagement with their healthcare and treatment. However, to date, there is little research on telemedicine and its impact on patient engagement.^49^ This is particularly made difficult because of the lack of conceptual clarity regarding patient empowerment versus engagement and activation. Moreover, there is little known about patient experiences, needs, or preferences. In an explorative qualitative study in Denmark, an app and workflow for follow-up were tested by 16 patients and 20 healthcare professionals and evaluated with interviews.^50^ The study showed that telemedicine improved patient reflection and collaboration, and patients felt more able to manage the posttransplant changes without additional burden (eg, because of the necessity to perform their own measurements). The app empowered patients in the consultation with their healthcare professional However, a few studies (outside transplant medicine) that have measured patient empowerment showed that there was no difference between telemedicine users and nonusers in patient activation and empowerment nor in patient satisfaction and also no change in patient empowerment over time.^51-53^ There is some evidence that enrollment in a self-monitoring program acts as a moderator of the relationship between patient activation and behavior change.^54^

## IMPACT OF THE CORONAVIRUS DISEASE 2019 PANDEMIC ON TELEMEDICINE FOR TRANSPLANT RECIPIENTS

Telemedicine, in general, has received a boost during the coronavirus disease 2019 (COVID-19) pandemic to provide acute home-based care not only for patients with COVID-19 but also for patients in need of continued chronic care during quarantine measures or staff shortages. Use of telemedicine before COVID-19 for chronic care may have been more common in rural areas; however, the COVID-19 pandemic has meant that telemedicine has been offered and made accessible to patients everywhere. The initiatives for telemedicine for transplant recipients with COVID-19 are limited to a few publications on a hand full of cases.^55-57^ In these articles, proof of concept was demonstrated, whereby telemedicine helped assess, diagnose, triage, and treat patients with COVID-19 while avoiding a visit to an emergency department or outpatient clinic. However, large-scale studies have not been published. The main effect of the COVID-19 pandemic seems to be a catalyst to speed up the development of telemedicine in regular care after kidney transplantation, as there was an urgent need to minimize the risk of infection, continuity of care, and ensure prompt interventions.^58^ A publication by the Italian group of Binda highlighted the need for a telemedicine program for kidney transplant recipients during the lockdown period as opposed to the standard follow-up by phone and email.^59^ Of interest is a publication by Chang et al who rapidly implemented a telehealth program during the first wave.^60^ In a letter to the editor, they report on 116 virtual visits among 108 transplant recipients, most (56%) done in recipients within 1 y of kidney transplantation, with 25% within the first 3 mo. This was successful, with only a minority (5%–10%) needing additional medical care after the virtual visits, although blood pressure or blood sugar testing for patients with diabetes was available during 74% and 59% of the visits, respectively, and many technical difficulties arose. The authors conclude that telemedicine offers a way to stay connected with patients, but the addition of mobile phlebotomy services and remote patient monitoring is essential for long-term virtual visits. Comparable experience with telemedicine and tips for rapid implementation have been published in other studies.^61,62^

As the pandemic continued, development of telemedicine also focused on psychosocial aspects related to COVID-19, like patient education, physical activity, and quality of life. During this period, patients relied more on web-based information, especially about COVID-19 and kidney transplantation. An analysis by van Klaveren et al^63^ showed that the educational quality of the information offered was limited to individual and passive learning, whereas group learning and active construction of knowledge were rarely encountered. The authors concluded that the educational quality of eHealth for transplant care needs to increase.

Although data on reduction in physical activity, specifically on transplant recipients, are lacking, they will undoubtedly have had decreased physical activity during the lockdown periods like most adults in the general population.^64^ However, in a multidisciplinary, multimodal, and telemedicine-based program (KTx360°-study; with among other things, a video chat supported endurance training 2–4 times per week)^65^ by Pape et al, the majority of the 248 transplant recipients (n = 136) reported no change in physical activity, whereas it improved in 80 patients and decreased in 32 patients during the first lockdown, compared with before the pandemic (*P* < 0.001).^66^ It remains unclear how much of these effects result from having ports therapy per se (with motivating physicians, mental health professionals, and sports scientists) or from the video-supported mode of delivery. Notably, during the lockdown period, this group strongly encouraged exercise and use of wearables, including a pedometer, and launched a Youtube channel with short educative clips. Nonetheless, using all available technologies seems to make a difference. A recent review supports this notion, showing that the use of physical activity monitors is safe and effectively increases moderate to vigorous physical activity, although the evidence was strongest for healthy individuals, and transplant recipients were not studied.^67^

## BARRIERS TO TELEMEDICINE FOR TRANSPLANT RECIPIENTS

Despite the many advantages of adherence, reduced travel time and cost, and provision of care, most publications also report challenges. These include technological limitations, digital literacy, patient willingness, the way the healthcare provider implements the service, legislation, policy, and financial burden (see Table 1).

### Availability, Language, and eHealth Literacy

In 2013, McGillicuddy et al^10^ described several limitations, including technological adeptness. Additionally, only 35% owned a smartphone at that time. A study 3 y later by the same group saw smartphone ownership rise to 61%, especially in younger transplant recipients, <55 y of age (75% versus 46% among those >55 y).^12^ These percentages will probably have risen over the past years because now 89% of the population in The Netherlands use smartphones on a daily basis.^68^ Notably, this percentage drops to 29 in the age group >75 y of age. Similar percentages are seen in other countries, like the United States, where >85% of adults own a smartphone.^69^ A phone survey from 2016 to 2017 among 178 kidney transplant recipients (and 110 liver transplant recipients) of 2 large American transplant centers showed that home internet access (92%) and mobile internet access (83%) were both high in a population with an average age of 52 y. Despite these high numbers, health literacy differed greatly among recipients. This trial was designed to evaluate differences in health literacy among frequent users of the hospitals patient portal (45% of recipients) compared with nonusers (18% of recipients) and showed significantly higher eHealth literacy in frequent users (32 versus 28 points on eHealth Literacy Scale; *P* < 0.001).^70^ Scores were also higher among younger transplant recipients, those who received college education, and those who had access to mobile internet but were not related to other sociodemographic characteristics, including race. This is in contrast to a recent trial, evaluating the effect of a sun-protection education on tablet computers in 170 kidney transplant recipients, in which health literacy was ascertained by a written self-administered survey in Spanish or English.^71^ In 28% of transplant recipients, health literacy was inadequate, especially among Hispanic Latino (94%) and non-Hispanic Black (75%) recipients.^72^

Research in nontransplant recipients confirms that patients’ health literacy as measured by the eHealth Literacy Scale significantly influenced adoption and use of telemedicine technology because 65% of patients with high eHealth literacy supported the adoption of a patient portal versus 38% of those with low eHealth literacy.^73^ Over the past decades, patients have been getting more familiar with technology and internet use, which makes education for the use of telemedicine applications easier. This has been demonstrated, for example, in The Netherlands, where the percentage of digital illiteracy decreased in the past 5 y.^68^

Illiteracy is another barrier that has insufficiently been addressed in current research. Illiterate transplant recipients are generally excluded from participating in studies.^68,72,73^ The illiterate recipient is underrepresented in the current literature of telemedicine, and the potential impact of telemedicine for this group is not yet known. Therefore, transplant care providers should make an effort to ensure that telemedicine is inclusive and that the benefits are available to every kidney transplant recipient, including the elderly, through education and training.

### Patient Willingness and Attitudes Toward Telemedicine

Another challenge of using new technology is the willingness of patients to engage and attitude toward technology. Willingness is likely being influenced by perception of burden (although this should be offset against the burden of the alternative of traveling to the healthcare center). In The Netherlands, just below half of the (healthy) elderly (average 75 y) had no intention of using medical applications.^74^ Among the significant factors in the decision-making of use were perceived usefulness and perceived ease of use. Table 2 summarizes the current data on telemedicine in kidney transplant recipients regarding patients’ willingness and attitudes.^10,12,50,75-78^ The study by McGillicuddy et al showed that approximately 80% of transplant recipients have a positive attitude toward telemedicine. This is confirmed by a recent survey by Reber et al,^75^ in which younger and higher educated recipients had higher affinity with telemedicine.

**TABLE 2.** Studies focused on attitude of kidney transplant recipients toward telemedicine and barriers, in chronological order

Author	Year	No. of patients	Design/objective	Results	Concerns
McGillicuddy et al^10^	2013	99	Patients attitude toward mobile phone–based remote monitoring and management of their medical regimen	79% (78/99) reported a positive attitude toward the use of the system Offers improved self-efficacy and improved medical management	Only 7% knew of the opportunity to use a mobile device for monitoring their medications 35% owned a smartphone Costs Technological adeptness
Browning et al^12^	2016	139	Cross-sectional study (questionnaire) to assess smartphone ownership, use of mHealth apps, and willingness to use it	61% owned a smartphone, 30% had prior knowledge of mHealth, and 7% were already using a mHealth app; 78% reported a positive attitude Younger patients (<55 y) were more likely to own a smartphone (75% vs 46%) and more frequently agree with use of mHealth (62% vs 36%)	Selection bias by recipients attending outpatient clinic
Reber et al^75^	2018	109	Cross-sectional study on the mobile technology affinity of kidney transplant recipients	57% used a smartphone or tablet and 45% used apps Younger and higher educated patients had significantly higher scores in mobile technology affinity	No data on internet use or reason for nonusage of smartphone/tablets/apps No data on attitude toward medication adherence app
Vanhoof et al^76^	2018	122 (30 kidney)	Cross-sectional, descriptive study in adult heart, lung, liver, and kidney transplant recipients to understand recipients’ overall willingness to use telemedicine for self-management support and investigate associations with relevant technology acceptance variables, and explore recipients’ views on telemedicine	Recipients rated importance of telemedicine for self-management on average as 7 on a 10-point numeric scale; higher educated patients, and users of telecommunication technology gave higher scores compared with lower educated or telecommunication nonusers Recipients preferred automatic data transfer, visual aids over text messages, and personal influence in access to telemedicine	Only 28% owned a smart phone (study performed in 2013) Most recipients were not eager to receive messages or reminders Selection bias by exclusion nonnative (Dutch) speakers
O’Brien et al^77^	2019	165	Cross-sectional study (questionnaire) to explore characteristics of users, use, barriers, and perceptions of mobile apps for self-management	Knowledge was greatest barrier reported by non–app users. Significantly fewer hospitalizations in mHealth app users versus other apps or nonusers (adjusted for patient demographics)	Selection bias by patients attending outpatient clinic Cross-sectional nature lacking causal relationship with hospitalizations
Nielsen et al^50^	2020	16	Explorative qualitative study on patients’ experiences of using a telehealth solution developed to improve follow-up after kidney transplantation	Transplant recipients found the app easy to use, and it facilitated support and management of problems. It improved preparation for consultation, improved dialogue, and enabled teleconsultation	Two training sessions were provided, possibly boosting adherence and usage of app 16 of 28 patients completed the test period No video consultations were possible but were desirable
Huuskes et al^78^	2021	34	Focus groups comprised 10 participants from kidney transplants recipients who joined via Zoom to have discussions on patient perspectives on telehealth during the COVID-19 pandemic	Different themes recognized, including minimizing burden (convenience and ease, efficiency of appointments, reducing exposure to risk, limiting work disruptions, and alleviating financial burden); attuning to individual context (respecting patient choice of care and ensuring a conducive environment); and empowerment and readiness (increased responsibility for self-management, confidence in physical assessment, mental preparedness, and forced independence)	Personal connection and trust needs protection. Hampering honest conversations, Less reassurance of follow-up and missed opportunity to share live experience Technical challenges and patient digital literacy

COVID-19, coronavirus disease 2019.

A cross-sectional study at the University Hospital Leuven, Belgium, among transplant recipients revealed a general willingness to use interactive health technology.^76^ This study also highlighted clear preferences such as automatic data transfers, use of visual aids (graphs) above text messages, personally deciding when to access the technology (instead of receiving reminders), and the preference for computers and the internet over smartphones. However, this study was done in 2013, and in the study, only 28% of transplant recipients possessed a smartphone. Given the now more frequent use of smartphones, some of these preferences might have changed, and more recent evidence on preferences is needed.

In 2017, in The Netherlands, a web-based self-management support system to support care for transplant recipients was evaluated. They received a point-of-care creatinine meter and a blood pressure monitor. During the first y posttransplantation, 54 patients registered their self-measured creatinine values in a web-based self-management support system that provided automatic feedback on the registered values (eg, to seek contact with a hospital). However, this program was hampered by the fact that kidney function had to be registered into the system by patients themselves, which they had a tendency to frequently postpone.^79^ Further analysis of this study showed that patients were on average positive toward using the self-management support system, especially if patients felt a positive effect toward the system.^80^

A more recent survey in 2020 focused on preferences of kidney transplant recipients for a mobile health application. This study revealed 3 themes, namely health tracking (medication, nutrition, fluid intake, laboratory values, and activity), feedback (short personalized messages, positive awards using symbols, and color-coded bar graphs indicating normal and abnormal ranges), and usability of the application itself (large fonts, words that everyone can understand, and all information stored in 1 area).^81^

In sum, for optimal development and implementation of telemedicine in transplantation, patients’ preferences, perceived burden, and needs have to be explored and integrated into technology design. A potential positive consequence of the COVID-19 pandemic is the increased willingness among transplant recipients to try new models of care delivery.^62^ Adding too many parameters or functions may in itself become a barrier to use or adherence, which has to be explored as well. A report about the experience of 15 kidney transplant recipients by Norwegian nephrologists confirmed the technical difficulties/deficiencies encountered by both patients and healthcare providers, but the majority of the patients were satisfied with this way of consultation.^24^ Of interest, facilitators of success were having a stable health condition and an established, trusting relationship with their nephrologist.

### Healthcare Center and Provider Challenges of Telemedicine

To start with a telemedicine program, the transplant center first needs to have the right infrastructure to provide digital care. This involves acquisition of technology and equipment that are preferably integrated into or at least compatible with current electronic medical records systems. The healthcare center would need to ensure continuous service availability with preferably 24-h back-up, adequate security and facilities, and personnel for telemedicine and home monitoring. The Achilles heel, in providing care at a distance, remains the ordering, cost, and availability in the electronic medical record of (blood) tests and the reliance on local healthcare providers (instead of patients themselves) to measure blood pressure, weight, or other values.

Another challenge for the healthcare provider is to adjust to a new way of providing healthcare. Healthcare providers need to become familiar with the technology, gain skills and confidence to use the technology, and integrate the technology into standard practice. This will require educational programs and support in case of problems. During this process, support should be readily available and surveys should be performed to monitor the experience of this new way of providing transplant care.^82,83^

There are a number of other considerations when initiating telemedicine in a transplant center. Recently, a group of Italian surgeons and nephrologists wrote a consensus paper on how to develop a model of video consultation for the regular follow-up of kidney transplant recipients.^56^ Eligible patients (or their caregivers) should have the skills in the use of electronic and mobile devices and be familiar with video call applications. Furthermore, the eligible patients should be stable in terms of both graft function and immunosuppressive regimen, which is a good starting point for any beginning telemedicine at a transplant center. Education of those not included should be started as well to grow the group eligible for telemedicine. A recent study in Finland on the implementation of a telemedicine program also highlights that the implementation of new systems should be started ahead of time, and the whole process must be well planned to achieve the desired final purpose.^84^

### Legislative Aspects and Responsibility of Data

In a recent editorial, Segev et al^85^ described in detail the effect of regulatory relaxations during the COVID-19 pandemic and how this should evolve to stimulate adoption of telemedicine in kidney transplantation. Although mostly specific to the United States, some aspects of legislative barriers apply to other countries with other systems as well. The acknowledgment that telemedicine is in fact comparable with in-person care and should be reimbursed accordingly (as stated above) is crucial to transform transplant care. Another important aspect of telemedicine recognized by Segev et al is patient privacy. Combined with the collection and security of the data, they must be protected in accordance with national and international law. As the data will be sent from the hospital to the patient and maybe a third-party server, the responsibility for the data and their safekeeping should be clear. How data will be transferred, where they will be saved, and how the data are collected and by whom are all challenges that need to be addressed thoroughly. It is important that there is trust in the system with regard to data collection and sharing, as shown by focus group meetings of kidney transplant recipients.^78^

### Financial Aspects of Telemedicine

Another major challenge is the financing of telemedicine. Cost and cost-savings for the use of telemedicine should be considered on all levels, from equipment needed to perform measurements, logging on to data servers, analyzing results, and giving support. The problem with reimbursement was also addressed in a German study.^34^ In their telemonitoring program, reimbursement had to be applied by the healthcare organization for on a case-by-case to over 100 different healthcare insurance companies, severely hampering implementation. There needs to be a sustainable business model to continue offering telemedicine services. As an example, the current reimbursement model in The Netherlands is based on physical consultations; therefore, if telemedicine is successfully implemented, fewer patients will visit the hospital, resulting in loss of income for the healthcare center. Setting up any new infrastructure requires a massive investment, which will be spread over patients over the first few years. Moreover, patients themselves need equipment at home and may not be willing or able to pay for equipment themselves. Insurance and government policies need to be adapted for telemedicine to make it a sustainable option for healthcare providers for continued use.^86^

## FUTURE DIRECTIONS

Whereas most initiatives for telemedicine seem to tackle only specific topics (like adherence or home measurements), ideally, an integrated, all-encompassing technological solution is required to fully transform digital care provision. Such an approach has been initiated by the group of Budde with their Medical Assistant for Chronic Care Service platform.^87^ In their publication, supported by an illustrative explanatory video, they presented this integrated solution. The Medical Assistant for Chronic Care Service platform enables transplant recipients to provide vital signs, well-being, and medication intake via smartphone apps. This information is transferred directly into a database and electronic health record and used for routine patient care via either medical messaging or video consultation. Physicians can also securely send an updated medication plan and laboratory data. This platform not only communicates between recipient and transplant center but also with the recipients’ local nephrologist. A telemedicine team reviews all incoming data and takes action if required. To date, the published results discussed the enrollment of 131 transplant recipients, and the effects of this integrated platform on care is eagerly awaited as it can become a blueprint for other transplant centers. Another initiative with an integrated approach is the KTx360°-study by Schiffer et al^65^ who focused on long-term improvement in posttransplant recipient management by the introduction of eHealth elements and additional integrated therapeutic options.

For blood sampling, methods like DBS still lack immediate results compared with measurements done in healthcare centers.^47,88^ Ideally, blood tests or even analysis should be done at home and shared with the healthcare professional. DBS gives the ability to perform multiple measurements at home, aiding in a more reliable measurement of exposure (eg, an area under the curve calculation) of the drug in question. With these results available to the patients, the next step would be feedback and self-adjustment of immunosuppressive drug dosage, aided by a dosing algorithm. Such algorithms are already developed and being tested in transplant recipients.^89,90^

As previously stated, home-based point-of-care creatinine measurements are available, but given their suboptimal diagnostic accuracy, other ways of measuring kidney function are under evaluation, for example, measuring creatinine or cystatin C in saliva. These techniques will also have to be validated for home application and integrated successfully into a monitoring loop with the healthcare provider.^47,91,92^

When an integrated, extensive telemedicine program has been successfully implemented and embraced by transplant recipients, telemedicine can move on from supporting the transplant care provider to becoming a program that fully supports the self-sufficiency of the transplant recipient, comparable with other fields of medicine. Diabetics adapt their insulin levels based on blood glucose levels guided by parameters calculated by their flash glucose measurements software. Most patients with heart failure self-monitor symptoms and vital signs and adjust their dose of diuretics when gaining too much weight and contact their healthcare provider if this approach fails. Their remote care often includes elements of patient education, counseling, and social and emotional support.^93^ In the future, we expect that transplant recipients will be able to become more self-sufficient in their treatment and possibly certain patient groups may only reach out to professionals in case of problems. Studies are needed to investigate the extent to which transplant recipients are willing and able to achieve such a high degree of self-sufficiency through telemedicine and the openness of professionals to this approach.

## CONCLUSION

With smartphones and internet access becoming more common among transplant recipients, telemedicine as part of routine care has become a serious option. Before the COVID-19 pandemic, the benefits of telemedicine had been demonstrated in multiple studies for both patients and healthcare providers; however, uptake and implementation were not universal. The pandemic paved the way to reduce 2 main barriers to telemedicine: patient willingness and reimbursement by insurance companies. This impetus now needs to be harnessed, and implementation may not be swift or easy. Successful implementation will take investment of time, effort, and resources. Patients’ preferences and needs have to be explored and integrated into every telemedicine program. Solutions are needed to overcome barriers to equal access so that all transplant recipients can benefit from the advantages of telemedicine. Now is the time for telemedicine to be integrated into standard transplant care, with a view to a future whereby transplant recipients will be more self-sufficient while receiving high-quality care at home.
